# Comparison of quantitative measurements between two different intravascular ultrasound catheters and consoles: in vitro and in vivo studies

**DOI:** 10.1007/s12928-021-00759-6

**Published:** 2021-02-27

**Authors:** Hiroyuki Okura, Makoto Watanabe, Akihiro Miura, Muneo Kurokawa, Tomoya Ueda, Tsunenari Soeda, Yoshihiko Saito

**Affiliations:** 1 Department of Cardiovascular Medicine, Nara Medical University, Kashihara, Japan; 2 Department of Cardiology, Gifu University Graduate School of Medicine, Gifu, Japan; 3 Department of Medical Engineering, Nara Medical University, Kashihara, Japan

**Keywords:** Coronary artery disease, Intravascular ultrasound, Atherosclerosis, Stent

## Abstract

Previous studies suggested possible discordant quantitative measurements between different IVUS catheters and/or systems. The purpose of this study was to assess compatibility of two different IVUS catheters and consoles for quantitative measurements of coronary arteries. (1). In vitro study: IVUS imaging was performed in a concentric cylindrical phantom with 6 sections of known, cross-sectional diameter ranging from 3.0 to 8.0 mm. The lumen diameter (LD) and lumen cross-sectional area (LA) were measured and compared. To compare between 2 different IVUS consoles, IVUS images were obtained using a single IVUS catheter (catheter 1) connected to 2 different IVUS consoles (console 1 and 2). To compare between 2 different IVUS catheters, IVUS imaging was obtained using 2 different IVUS catheters (catheter 1 and 2) connected to a single IVUS console (console 2). (2). In vivo study: IVUS imaging was performed in 40 stented coronary arterial segments from 40 patients. The maximal stent diameter (Max SD), minimal stent diameter (minSD), and stent area (SA) were measured at both distal and proximal stent edges and compared between the two IVUS consoles (console 1 and 2) connected to a single IVUS catheter (catheter 1) (*n* = 20). IVUS imaging was also performed to compare between catheter 1 and 2 connected to IVUS console 2 (*n* = 20). Both in vitro and in vivo studies showed good correlation between the two IVUS consoles as well as two IVUS catheters. In conclusion, two IVUS catheters and consoles provide comparable IVUS measures both in vitro and in vivo.

**Supplementary Information:**

The online version contains supplementary material available at 10.1007/s12928-021-00759-6.

## Introduction

Intravascular ultrasound (IVUS) is widely used to guide percutaneous coronary intervention (PCI) [1–5] and to assess efficacy of medication or PCI on coronary atherosclerotic plaque or stent in vivo [6–8]. Previously, there were discordance in quantitative measurements of the coronary arteries using different IVUS systems from different providers mainly because of the differences in the speed of sound used in each IVUS system [9–11]. The aim of this study was to assess compatibility of two different IVUS catheters and consoles for quantitative measurements of coronary arteries.

## Methods

### In vitro study

**Fig. 1:**
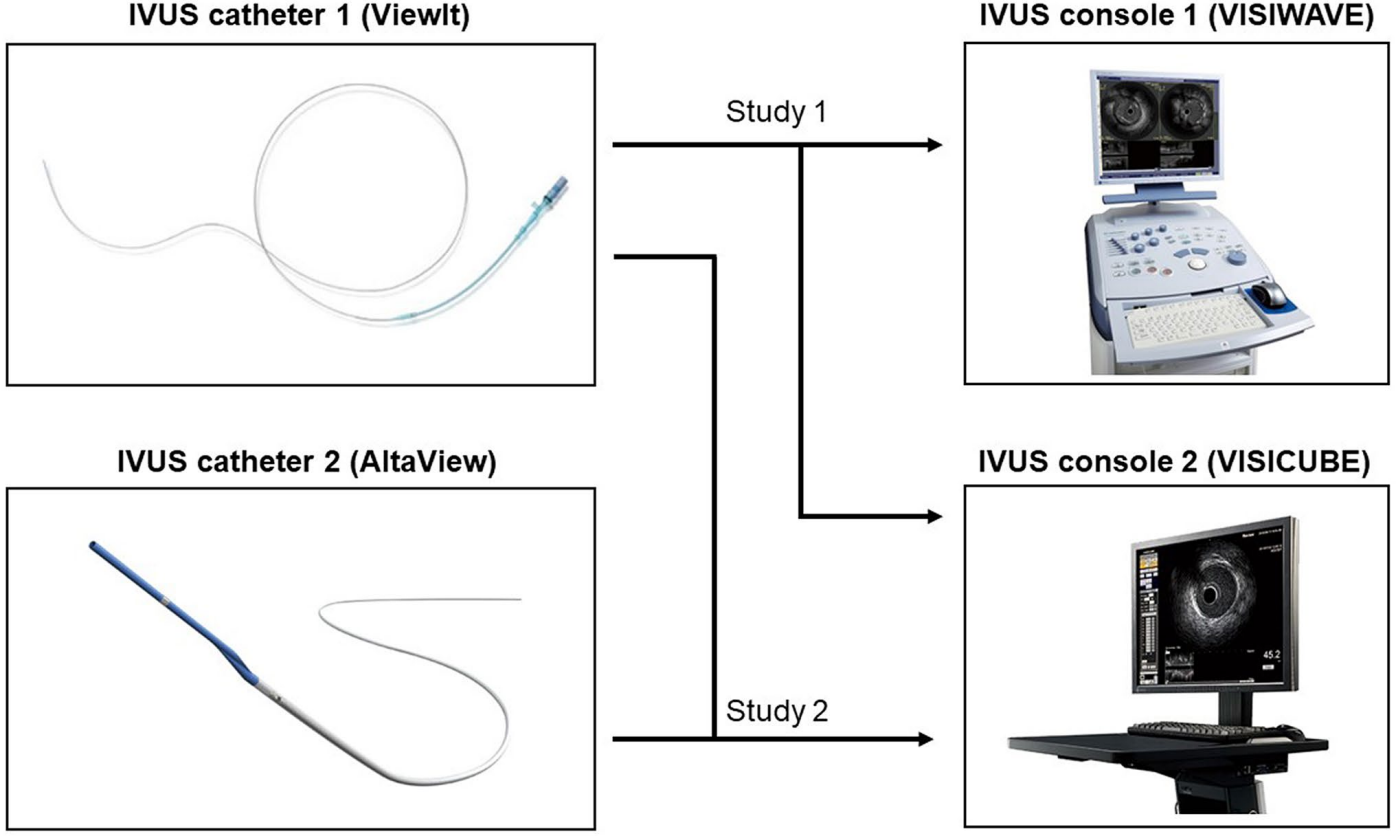
IVUS catheters and consoles used in this study. Study 1 compared 2 consoles and study 2 compared 2 catheters

IVUS imaging was performed in a concentric cylindrical phantom with 6 sections of known, cross-sectional diameter ranging from 3.0 to 8.0 mm as previously reported [12]. Images were obtained in a saline-filled tank maintained at 37 ◦C. Commercially available IVUS catheters with a mechanical rotating 40 MHz transducer (catheter 1) (ViewIT™, Terumo Corporation, Tokyo, Japan) or 60 MHz transducer (catheter 2) (AltaView™, Terumo Corporation, Tokyo, Japan) were used in this study. IVUS imaging was recorded using catheter 1 connected to either IVUS console 1 (VISIWAVE™, Terumo Corporation, Tokyo, Japan) or an IVUS console 2 (VISICUBE™, Ueda Japan Radio Co. Ltd, Nagano, Japan) to compare two IVUS consoles (Fig. 1, study 1). IVUS catheter was inserted sequentially into the distal edge of the tube and slowly pulled back automatically to the proximal edge. All procedures were performed by the same operator for both systems. In each tube, 5 arbitrary segments were selected for analysis. The lumen diameter (LD) and lumen cross-sectional area (LA) were measured and compared. Then, IVUS imaging was performed using IVUS catheter 2 (AltaView™) connected to console 2 (VISICUBE™) to compare two IVUS catheters (Fig. 1, study 2).

### In vivo study

Forty patients with native coronary artery disease who underwent stent implantation were enrolled in the in vivo study. The first 20 patients underwent IVUS imaging after PCI by IVUS catheter 1 (ViewIT™) connected to either IVUS console 1 (VISIWAVE™) or IVUS console 2 (VISICUBE™) to compare two IVUS consoles (Fig. 1, study 1). The next 20 patients underwent IVUS imaging after PCI by IVUS catheter 1 (ViewIT™) followed by IVUS catheter 2 (AltaView™) connected to console 2 (VISIWAVE™) to compare two IVUS catheters (Fig. 1, study 2). Images with a significantly reduced quality were excluded. This study was in compliance with the Declaration of Helsinki with regard to investigations in humans, and the study protocol was approved by the Ethics Committee of Nara Medical University Hospital (Number 1278 and 1754). Written informed consent was obtained from all patients before cardiac catheterization and IVUS imaging.

### IVUS imaging and analysis

IVUS pullback was performed using an automated pullback device at a rate of 0.5 mm/s or 9.0 mm/s (IVUS catheter 2 only). The IVUS images were continuously recorded on each IVUS console and then transferred to CD-ROM for offline analysis. The IVUS images recorded on CD-ROM were analyzed using a commercially available planimetry system (VISIATLAS™, Terumo Corporation, Tokyo, Japan) [13]. In the in vivo study, coronary stented segments were selected, and images of the proximal edges and distal edges were selected for cross-sectional measurement. Maximal stent diameter (MaxSD), minimal stent diameter (MinSD), and stent area (SA) were measured at both distal and proximal stent edges (both study 1 and 2). Using an automated pullback device at a rate of 0.5 mm/s (both catheter 1 and 2) or 9.0 mm/s (catheter 2 only), total stent length (TSL) was measured and compared between the 2 systems (study 2).

### Statistical analysis

Statistical analysis was performed with StatView version 5.0 (SAS Institute, Cary, NC, USA). Sample size was determined based on the previous similar studies [10, 12]. Continuous variables are reported as mean ± SD. The comparisons between two different IVUS catheters or consoles were performed using linear regression analysis. A *P* value of < 0.05 was considered to be significant.

## Results

### In vitro study

Both LD and LA obtained by the two IVUS systems correlated well with the actual size of the tube (Table 1, Fig. S1). Comparison between two different IVUS catheters is shown in Table 2. Two different catheters provide similar quantitative measures. Comparison between two different IVUS consoles is shown in Table 3. IVUS measurements obtained by a single IVUS catheter connected to two different IVUS consoles provide similar results.

**Table 1:** Comparison between IVUS measurements and true value (in vitro study)

Catheter	Console	Measurements	Correlation formula	*R*	*P* value
ViewIT	VISIWAVE	LD	*Y* = 1.00 *X* + 0.05	0.9999	< 0.001
		LA	*Y* = 0.99 *X* – 0.27	0.9999	< 0.001
ViewIT	VISICUBE	LD	*Y* = 1.00 *X* – 0.02	0.9999	< 0.001
		LA	*Y* = 1.01 *X* – 0.18	0.9999	< 0.001
AltaView	VISICUBE	LD	*Y* = 1.01 *X* – 0.11	0.9998	< 0.001
		LA	*Y* = 1.00 *X* – 0.57	0.9998	< 0.001

*LA* lumen area, *LD* lumen diameter

**Table 2:** Comparison between 2 different IVUS catheters (in vitro study)

Catheter 1	Catheter 2	Console	Measurements	Correlation formula	*R*	*P* value
ViewIT	AltaView	VISICUBE	LD	*Y* = 1.00 *X* + 0.05	0.9999	< 0.001
			LA	*Y* = 0.99 *X* – 0.27	0.9999	< 0.001

*LA* lumen area, *LD* lumen diameter

**Table 3:** Comparison between 2 different IVUS consoles (in vitro study)

Catheter	Console 1	Console 2	Measurements	Correlation formula	*R*	*P* value
ViewIT	VISICUBE	VISIWAVE	LD	*Y* = 1.00 *X* – 0.03	0.9998	< 0.001
			LA	*Y* = 1.01 *X* – 0.21	0.9997	< 0.001

*LA* lumen area, *LD* lumen diameter

### In vivo study

**Fig. 2:**
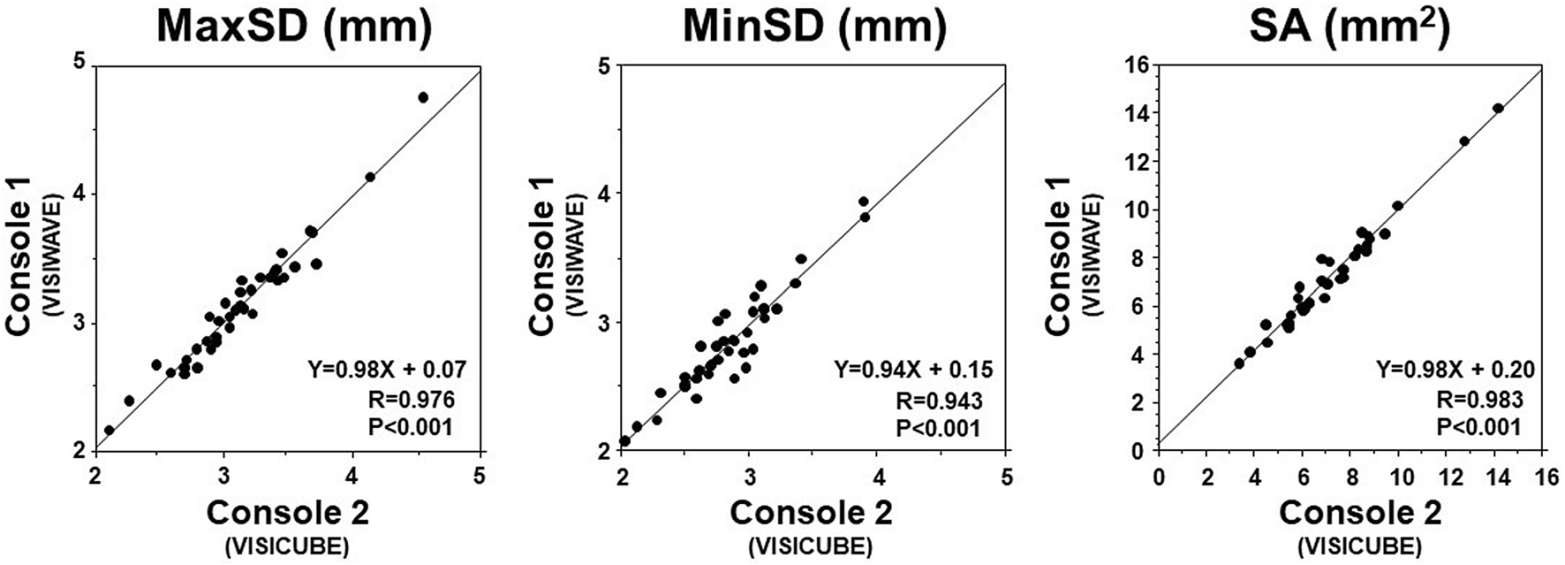
Comparison between 2 different IVUS consoles (in vivo). MaxSD, MinSD, and SA showed good correlations between IVUS console 1 and 2

**Fig. 3:**
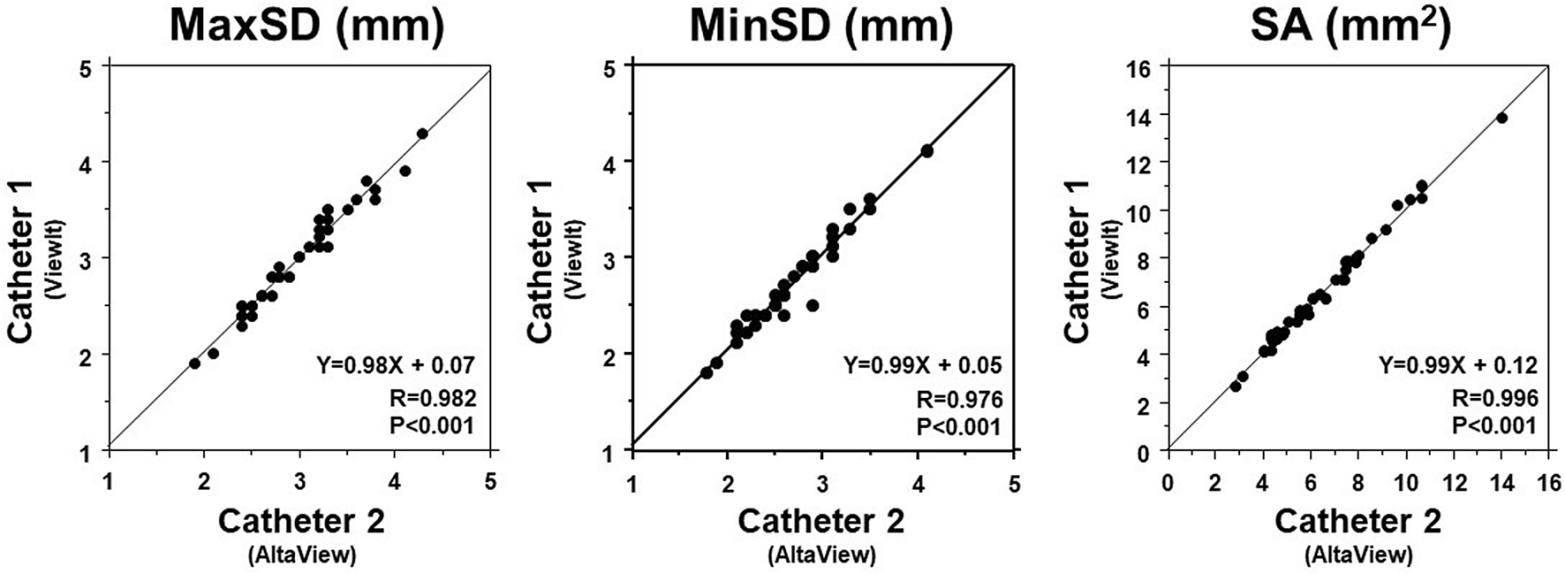
Comparison between 2 different IVUS catheters (in vivo). MaxSD, MinSD, and SA showed good correlations between IVUS catheter 1 and 2

Figure 2 shows the correlation between the values from catheter 1 (ViewIT™) with console 1(VISIWAVE™) vs. catheter1 (ViewIT™) with console 2 (VISICUBE™). MaxSD (*R* = 0.976, *P* < 0.001), minSD (*R* = 0.943, *P* < 0.001), and SA (*R* = 0.983, *P* < 0.001) obtained by the two IVUS consoles have a good correlation. Figure 3 shows the correlation between the values from catheter 1 (ViewIT™) with console 2 (VISICUBE™) and catheter 2 (AltaView™) with console 2 (VISICUBE™). MaxSD (*R* = 0.982, *P* < 0.001), minSD (*R* = 0.976, *P* < 0.001), and SA (*R* = 0.996, *P* < 0.001) obtained by the two IVUS catheters connected to the same console (console 2) have a good correlation.

**Fig. 4:**
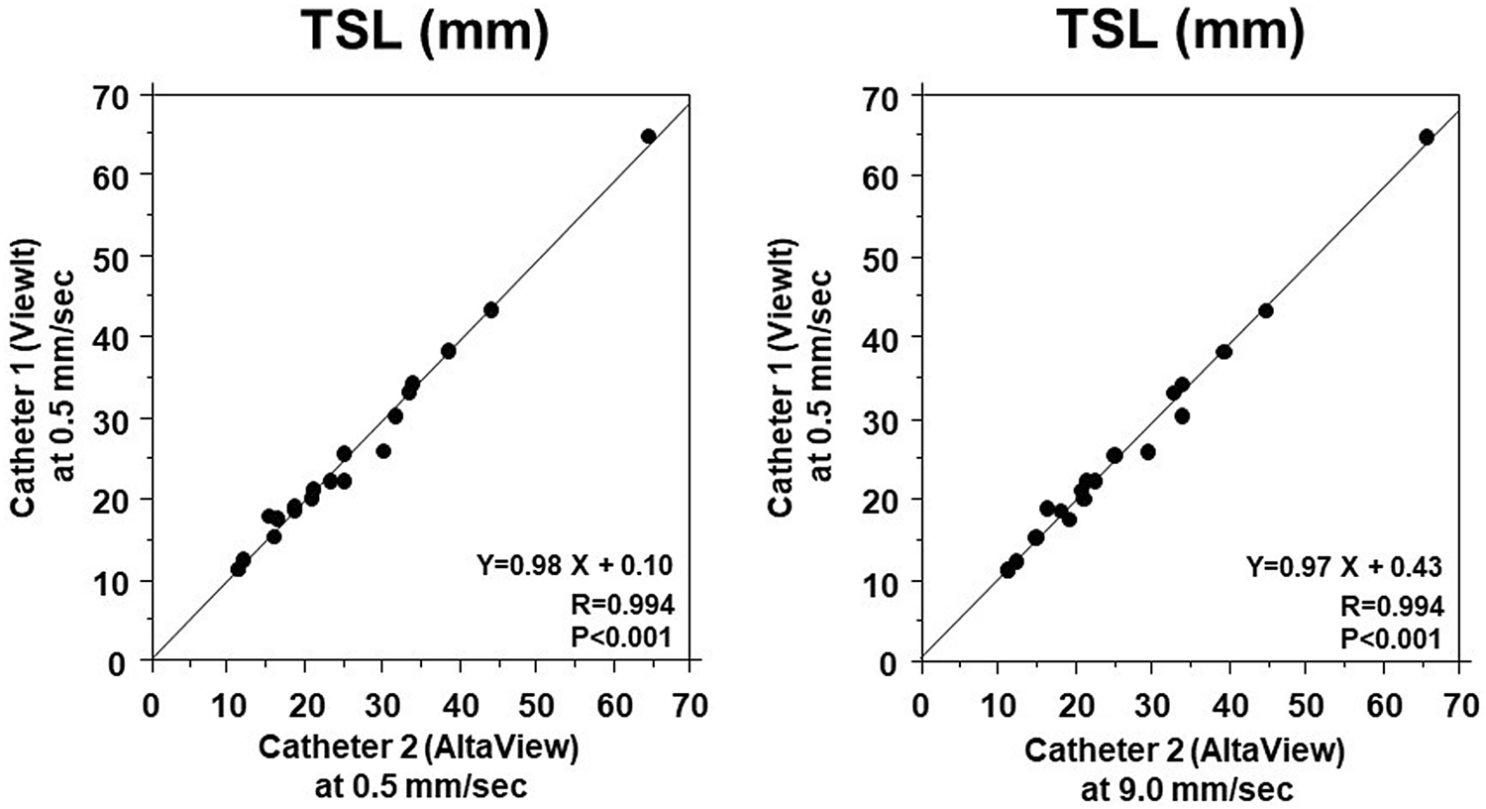
Comparison of IVUS-derived total stent length (TSL) between 2 catheters. TSL obtained by IVUS catheter 1 at a rate of 0.5 mm/s correlated well with TSL obtained by catheter 2 at a rate of 0.5 m/s (left panel). TSL obtained by IVUS catheter 1 correlated well with TSL by IVUS catheter 2 at a rate of 9.0 mm/s

IVUS-derived TSL measured by catheter 1 at a rate of 0.5 mm/s and catheter 2 at a rate of 0.5 mm/s showed a good correlation (*R* = 0.994, *P* < 0.001) (Fig. 4). Similarly, TSL by catheter 1 at a rate of 0.5 mm/s and catheter 2 at a rate of 9.0 mm/s showed a good correlation (*R* = 0.994, *P* < 0.001) (Fig. 4). In addition, TSL by catheter 1 at a rate of 0.5 mm/s and catheter 1 at a rate of 9.0 mm/s showed a good correlation (*Y* = 1.02X − 0.46, *R* = 0.996, *P* < 0.001, figure not shown).

## Discussion

Our study demonstrated that a new IVUS catheter as well as IVUS console provide accurate quantitative measurements. In vitro study demonstrated that a new IVUS catheter and IVUS console provide quantitative measures comparable to a previously validated IVUS catheter and IVUS console. In addition, two IVUS consoles with different pullback device provided comparable length measurements. These results suggest that currently available IVUS system can be used during PCI procedures as well as clinical research similar to the previous version of the IVUS catheter and console.

Currently, IVUS has been routinely used during PCI[3–5]. IVUS provides quantitative measures to select device size and to optimize PCI results [1, 4]. We do believe that IVUS-derived measurements are accurate and reproducible. However, early studies demonstrated discrepancies in quantitative results between different IVUS systems [9–11]. An in vitro study comparing between 40 MHz mechanical IVUS (Atlantis SR/Ultracross, Boston Scientific) and 20 MHz phased array IVUS (InVision/Avanar, Volcano, Rancho Cordova, CA, USA) demonstrated up to 18% differences in quantitative results between the 2 systems [9]. Similarly, an ex vivo human coronary artery study showed that plaque area and plaque burden were significantly larger in mechanical IVUS (Atlantis SR) than phased array IVUS (InVision)[11]. Even between similar IVUS systems (mechanical IVUS) (40 MHz Atlantis versus 35 MHz Intrafocus, Terumo), in vitro as well as in vivo study demonstrated that lumen diameter, lumen area, plaque plus media area, and external elastic membrane area by one IVUS (Intrafocus) were significantly smaller than those obtained by another IVUS (Atlantis)[10]. Difference in the speed of ultrasound used in each IVUS system (1530 m/s in Intrafocus and 1562 m/s in Atlantis) and the frequency of the transducer (35 MHz in Intrafocus and 40 MHz in Atlantis) are suspected as possible causes of the differences in quantitative measurements [12]. Because the second-generation IVUS catheters and systems used similar frequencies and the same a speed of sound, quantitative measurements between different IVUS systems should be comparable. Indeed, a previous study comparing two different IVUS systems from different companies demonstrated comparable quantitative measures [12]. Yamada et al. compared two different mechanical rotating IVUS catheters (Atlantis Pro2™, Boston Scientific Corporation, Natick, MA, USA and ViewIT ™, Terumo Corporation, Tokyo, Japan) and showed that these 2 different commercially available IVUS systems were accurate and comparable. Recently, a brand new IVUS catheter and console with higher frequency and higher pullback speed (AltaView and VISICUBE) has become clinically available. Although theoretically the new IVUS catheter and console would provide comparable quantitative measures, validation study is needed. As expected, our data confirmed that the new IVUS catheter and IVUS console provided accurate and comparable results compared with older IVUS catheter and IVUS console. Because ViewIT and VISIWAVE have not been completely replaced by the AltaView and VISWAVE, it is important to demonstrate the compatibility of either IVUS catheter or IVUS console. Indeed, ViewIT is still on the market and is used with either VISIWAVE or VISICUBE. In this regard, our results have strong clinical implications with respect of the reliability of the quantitative IVUS measures during IVUS-guided PCI. Furthermore, IVUS is also used to assess serial changes in coronary vessel, plaque as well as stent to evaluate efficacy of drug and stent itself [6–8, 14, 15]. Therefore, our results have implication, showing that quantitative data obtained from different IVUS catheters and consoles could be used.

Previous studies demonstrated that length measurements by IVUS using motorized pullback of the transducer and imaging core through a stationary imaging sheath provide accurate length and thus have been clinically utilized [16, 17]. These studies used constant pullback at a rate of 0.5 mm/s because of limited frame rate (30 frames /s). Because of the variable and higher frame rates (30–90 frames/s) used in the newer IVUS console (VISICUBE), IVUS imaging can be performed at variable and higher pullback speed (0.5–9.0 mm/s). Therefore, we also compared stent TSL between standard pullback speed (0.5 mm/s) and higher pullback speed (9.0 mm/s), and demonstrated that stent length measurements can be reliably performed using higher pull back speed at a rate of 9.0 mm/s. These results also have an important clinical implication during PCI. With use of fast pullback before PCI, we could avoid risk of ischemia during IVUS pullback and shorten procedure time. Although faster pullback speed may be advantageous for accurate measurements, because it is less affected by cardiac motion, our results do not indicate that one is better than the other. Larger sample size with variable vessels would be necessary to answer this question.

Our current study has several limitations. First, we compared between 2 catheters and consoles from a single company. Therefore, it is not conclusive that the newer IVUS catheter (AltaView) and a console (VISICUBE) provide similar quantitative data compared with another IVUS systems from different companies. Second, we only imaged and measured stented segment to minimize the risk of complications by performing multiple IVUS pullback imaging before treatment. Therefore, it is uncertain if pre-intervention IVUS-derived quantitative measures are comparable [2]. In particular, quantitative measurements, especially length measurements based on the IVUS pullback recording, may be affected by the tortuosity of the vessel. Results of our present study assessing the stented segments only may not be applicable to the tortuous vessel. Third, we performed only single IVUS imaging pullback recording with each IVUS catheter by a single operator; therefore, the impact of the IVUS imaging method such as order of catheter insertion or differences in insertion/recording technique by each operator could not be evaluated. However, it is unlikely that quantitative results are affected by them. Finally, qualitative assessment of the coronary plaque was not compared between the 2 catheters and 2 consoles, because this was not the purpose of this study. Comparison of visually assessed plaque types or IVUS-derived tissue characterization (IB-IVUS) would be required to answer this question [18–21].

In conclusion, a new version of IVUS catheter and console provide accurate and comparable quantitative measures as compared with a previous IVUS catheter and console. Therefore, these two catheters and consoles are exchangeable and could be comparably used during IVUS-guided PCI and IVUS-based clinical investigations.

## Supplementary Information

Fig. S1 Correlations between LD (upper panel) and LA (lower panel) obtained by ViewIt & VISIWAVE (left), ViewIt & VISICUBE (middle) and AltaView & VISICUBE (right) with actual size (phantom LD and LA). LD:lumen diameter; LA: lumen area.

Supplementary file1 Correlations between LD (upper panel) and LA (lower panel) obtained by ViewIt &VISIWAVE (left), ViewIt &VISICUBE (middle) and AltaView &VISICUBE (right) with actual size (phantom LD and LA). LD: lumen diameter; LA: lumen area (JPG 102 KB)
