# Supplementary figures and images for: Comparison of quantitative measurements between two different intravascular ultrasound catheters and consoles: in vitro and in vivo studies
Source: Cardiovasc Interv Ther. 2021 Feb 27;37(1):109–15. doi: 10.1007/s12928-021-00759-6 (PMC8789722; doi:10.1007/s12928-021-00759-6)

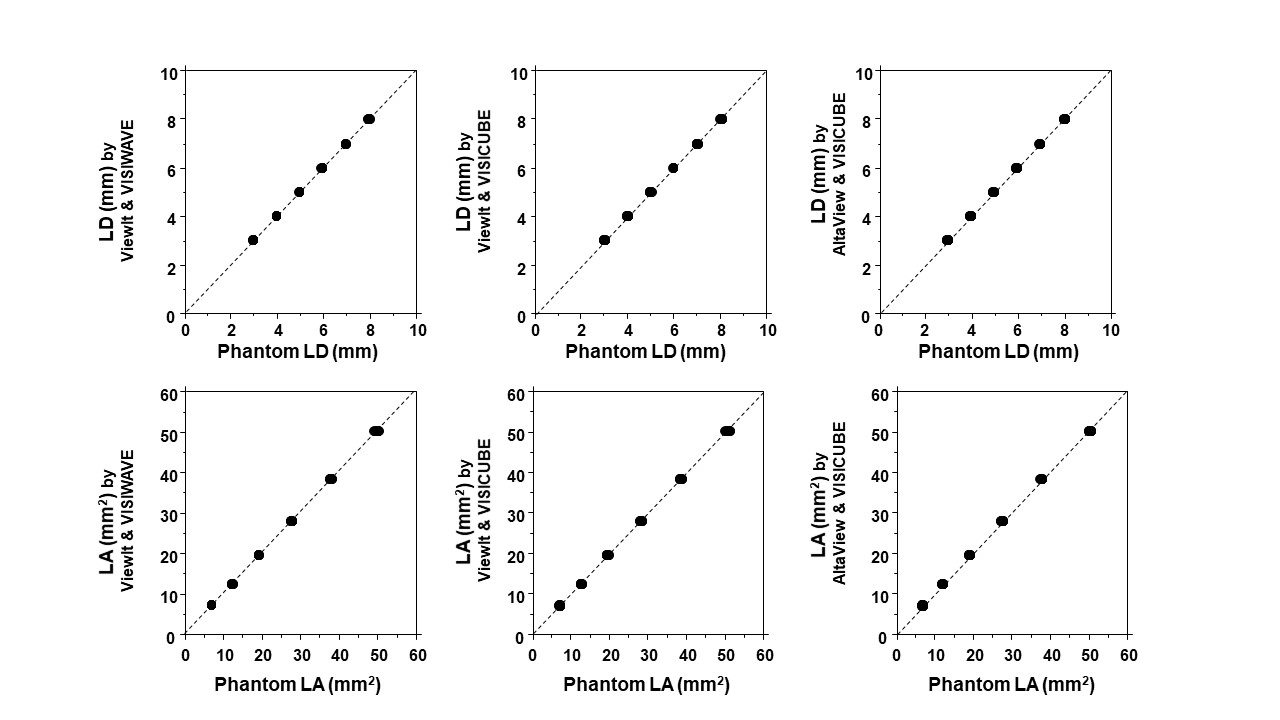

Supplement: Supplementary file 1 — Supplementary file1 Correlations between LD (upper panel) and LA (lower panel) obtained by ViewIt &VISIWAVE (left), ViewIt &VISICUBE (middle) and AltaView &VISICUBE (right) with actual size (phantom LD and LA). LD: lumen diameter; LA: lumen area (JPG 102 KB) [file 12928_2021_759_MOESM1_ESM.jpg]
